# The Level and Outcomes of Emotional Labor in Nurses: A Scoping Review

**DOI:** 10.1155/2024/5317359

**Published:** 2024-10-15

**Authors:** Hanbo Feng, Meng Zhang, Xueting Li, Yang Shen, Xiaohan Li

**Affiliations:** ^1^School of Nursing, China Medical University, Shenyang, China; ^2^Dermatology Department, Nanjing University Medical School Affiliated Nanjing Drum Tower Hospital, Nanjing, China

**Keywords:** emotional labor, nurses, nursing management, scoping review

## Abstract

**Aims:** This scoping review aims to examine the level and outcomes of emotional labor in nurses.

**Background:** Nursing is a highly emotional profession that requires nurses to experience a wide range of emotions and control their emotional expressions in nursing practice. Chronic emotional labor and emotional burden not only impact nurses' individual well-being and professional performance but also their job satisfaction and turnover intention.

**Evaluation:** This scoping review was guided by a five-stage scoping review framework and the PRISMA-ScR checklist. Eight databases were searched and 41 studies were reviewed.

**Key Issues:** The Emotional Labor Scale and the Emotional Labor Scale for Nurses were the most commonly used instruments. Significant associations were found between emotional labor and 52 outcomes, which were categorized into two main themes: nurses' personal well-being and organizational well-being. There were 19 mediators and 12 moderators between nurses' emotional labor and 26 different outcomes.

**Conclusions:** While the level of emotional labor among nurses remains uncertain, its consequences are plentiful and intricate. Studies have shown an important relationship between nurses' emotional labor and individual and organizational well-being, which is crucial for nurse managers. There is a need to explore the positive effects of nurses' emotional labor and its impact on patients. Additionally, validation studies of the instruments used to measure nurses' emotional labor are necessary.

**Implications for Nursing Management:** The insights gained from this scoping review provided a more comprehensive understanding of emotional labor for nurse managers. This knowledge can be utilized to design training programs on emotional management and to explore different interventions aimed at alleviating the adverse impacts of nurses' emotional labor.

## 1. Background

Emotional labor was proposed by Hochschild [1] in her book “The Managed Heart: Commercialization of Human Feeling.” It was defined as the management of feelings to create a publicly observable facial and bodily display for reward. A two-dimensional structure of emotional labor was proposed, namely, surface acting and deep acting [1]. Surface acting is when employees express emotions that differ from their true feelings by adjusting their physical behavior or facial reactions without shaping their inner feelings, such as faking or hiding emotions. Deep acting not only modifies their external emotional expression but also attempts to experience the displayed emotion through various techniques. For example, individuals may imagine positive emotional events to feel and express positive emotions. Subsequently, Diefendorff, Croyle, and Gosserand [2] verified that the expression of naturally felt emotions is a unique strategy in addition to surface acting and deep acting. It means that employees' authentic emotional experience is consistent with the emotional presentation rules required by the organization, and they will naturally express their genuine emotions. Since Hochschild's work, emotional labor has been widely applied in various fields, including nursing. Smith [3] introduced the concept of emotional labor to the field of nursing by describing how student nurses and ward sisters manage their emotions for the benefit of patients. Emotional labor in nursing is conceptualized as a process whereby nurses adopt a “work persona” to express their autonomous, surface or deep emotions during patient encounters [4].

Nursing is an emotionally complex occupation, and nursing work involves a combination of emotional, physical, cognitive, and organizational labor [5]. Given that care and patient-centered interpersonal orientation are central to the spirit of the nursing profession [6], nurses often need to regulate their emotions and exhibit appropriate emotional states during patient interactions [4]. Emotional labor is a high level of professional competence for nurses, and emotional involvement is an essential element of the therapeutic relationship between healthcare professionals and patients [7]. According to the conservation of resources theory, chronic emotional labor and emotional burden not only affect nurses' professional performance and job satisfaction but also their physical and mental health, as well as the quality of patient care [8].

Over the past few decades, numerous studies have focused on the outcomes of emotional labor for nurses, such as burnout, turnover intention, and job satisfaction. Despite these major findings, several issues deserve our attention. On the one hand, the research findings of nurses' emotional labor are varied, involving individual mental health [9], organizational commitment [10], work engagement [11], and service levels [12]. On the other hand, these findings have not always been consistent. For example, research has shown that nurses' emotional labor is positively associated with burnout and turnover intention [13]. However, Peng found a positive effect of nurses' emotional labor, which had a beneficial impact on their professional values [14]. Furthermore, there is a lack of synthesis of knowledge regarding the outcomes of emotional labor in nurses.

It is imperative for nurse managers to understand the level and outcomes of emotional labor. First, nurses experience a significant amount of emotional labor. 52.97% of nurses reported that they experienced empathetic emotions such as sympathy, sadness, compassion, and grief [15]. Additionally, nurses reported a 34.1% prevalence of emotional exhaustion during the COVID-19 pandemic according to a systematic review [16]. Second, emotional labor may have negative impacts on nurses and nursing organizations, such as affecting nurses' physical and mental health [17], decreasing nurses' work engagement [11], and increasing their burnout [18], which in turn affects the quality of patient care. Third, the shortage of nursing staff and high turnover rates have been major issues in global healthcare [19]. The study indicated that nurses' emotional labor was a significant factor contributing to their turnover intentions [20]. A better understanding of the level and consequences of emotional labor for nurses is essential for developing effective interventions. Nurse managers can implement strategies to prevent and manage burnout in nurses, as well as promote effective emotional management strategies for nurses. Therefore, the scoping review aims to evaluate and summarize existing data on emotional labor level and outcomes in nurses.

## 2. Review Questions

● What is the level of nurses' emotional labor and how is it measured in the existing literature?
● What outcomes for nurses and patients are associated with emotional labor, and what are the mediators and moderators between emotional labor and these outcomes?

## 3. Methods

### 3.1. Design

This scoping review was performed following the five-stage approach of Arksey and O'Malley: (1) identifying the research question, (2) identifying relevant studies, (3) study selection, (4) charting the data, and (5) collating, summarizing, and reporting the results [21]. The protocol was registered on the Open Science Framework. The PRISMA-ScR checklist [22] was used to guide this review.

### 3.2. Searching Strategy and Study Selection

Several electronic literature databases (Web of Science, PubMed, CINAHL, PsycINFO, Scopus, Embase, ScienceDirect, and Wiley Online Library) were searched to identify relevant studies until April 2024. We selected “nurs^∗^,” “emotional labour,” “emotional labor,” and “emotional work” as the search keywords by referring to the similar reviews [23, 24]. Studies were sought in English, and an example of the search strings in PubMed is provided in Appendix A, Supporting Data.

Relevant articles were considered for inclusion if they met the following criteria: (1) data from original studies, (2) related to nurses (publications discussing other healthcare workers or nursing students were excluded), (3) measured the level of emotional labor of nurses quantitatively, (4) reported personal or work-related outcomes related to emotional labor of nurses, (5) utilized regression analysis or path analysis to analyze the data, (6) the full text was available, (7) written in English, and (8) published in peer-reviewed journals.

All retrieved studies were imported into EndNote software. After removing duplicates, two researchers (HBF and XTL) independently reviewed the titles, abstracts, and full texts of the studies according to the pre-established criteria.

### 3.3. Data Extraction

Data from the included articles were extracted after a thorough review of the literature. A standardized Microsoft Excel spreadsheet was used for data extraction, including author, publication date, country/region, study design, study aim, population sample, theoretical basis, the tool used to measure emotional labor, emotional labor scores, outcomes, data analysis, and recommendations.

### 3.4. Data Synthesis

The extracted data were summarized narratively. Characteristics of the included studies, scales to measure emotional labor, and significant outcomes in quantitative studies were classified and presented in tabular form. The outcomes of emotional labor were described in terms of individual well-being and organizational well-being according to Grandey's emotional regulation model [25]. Individual well-being includes burnout and job satisfaction, and organizational well-being involves performance and withdrawal behaviors.

## 4. Results

### 4.1. Search Results

**Figure 1:**
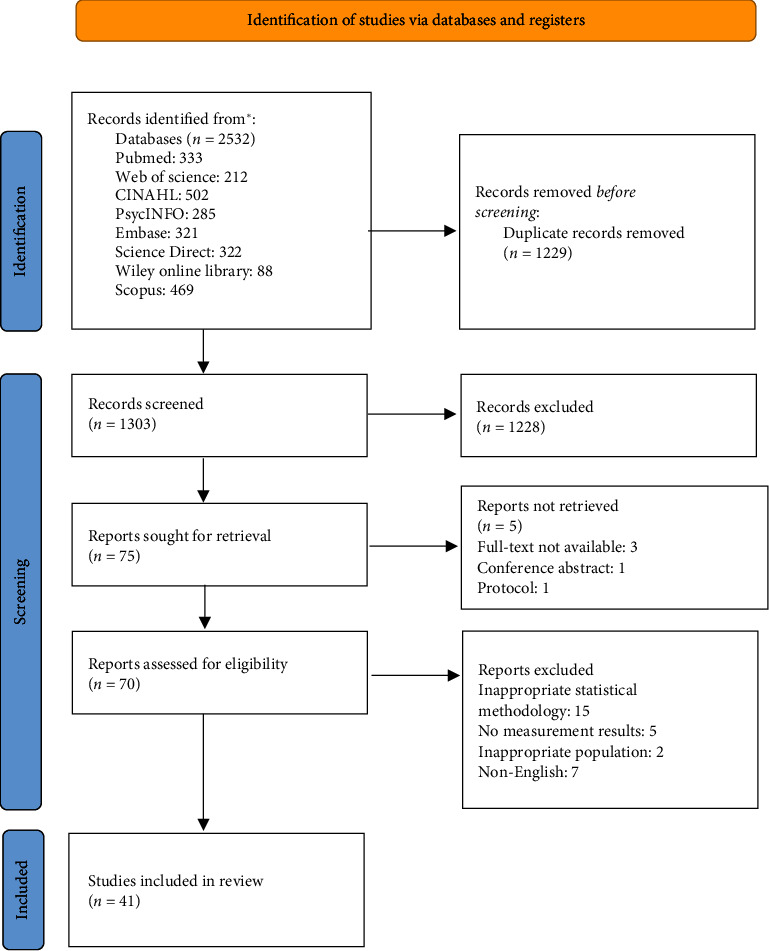
PRISMA flowchart for study selection.

We identified a total of 2532 studies up to April 2024. 1303 articles were retained after removing duplicates. Among these articles, we conducted two rounds of review: the first was based on the title and abstract, and the second aimed to remove articles that did not meet the inclusion criteria by reading the full text. In addition, we did not limit the publication time to maximize the search for relevant studies. After that, 41 studies were selected for the review.  Figure 1 shows the PRISMA flowchart of the whole selection process.

### 4.2. Study Characteristics

Table 1 shows the main characteristics of the included studies. 41 articles were published from 2011 to 2024, and 24 (58.54%) of the studies were published in the last five years. They used a cross-sectional study design (*n* = 39) and longitudinal design (*n* = 2). Most studies were conducted in East Asia, with 14 in Korea and 10 in China. Among the remaining 17, 6 were conducted in Australia, 3 in Turkey, 2 in the USA, 1 in Finland, 1 in Germany, 1 in Ghana, 1 in India, 1 in Iran, and 1 in Italy. The participants in 37 studies were general registered nurses, 2 studies included nurses and midwives, 2 studies included psychiatric nurses, and 1 study involved community nurses. The settings for all studies were hospitals. Regarding the theoretical framework of the included studies, 21 theories were used in 17 studies. The most commonly used theories were the Job Demands-Resources Model (*n* = 7) and the Conservation of Resources Theory (*n* = 5). All the findings presented were related to nurses' emotional labor. The statistical analysis methods used included linear regression, mediator model, and structural equation model.

### 4.3. Instruments for Measuring Emotional Labor

The Emotional Labor Scale developed by Brotheridge and Lee (*n* = 8) and the Emotional Labor Scale for Nurses (*n* = 6) were the most commonly used instruments to measure emotional labor in nurses. There were several other emotional labor scales developed from different researchers, for example, the emotional labor scale developed by Morris and Feldman and the emotional labor scale developed by Diefendorff.  Table 2 summarizes the properties of the instruments and the scores of emotional labor among nurses.

### 4.4. The Level of Emotional Labor Among Nurses

There were considerable variations in the scales measured, the range of scores (1 to 4, 1 to 5, 1 to 6, and 1 to 7), and the dimensions among the instruments used in the included studies. For example, the scale used by Karimi et al. [17] had only a single dimension of emotional dissonance. Bartram's et al. [18] study only provided the number of items on the scale without describing the score range and dimensions. Even though they used the same scale, some articles reported mean scores while others reported total scores. Therefore, it is difficult to compare the levels of emotional labor between studies.

Therefore, we used the descriptions in the studies to define the level of emotional labor of the nurses. Six studies reported emotional labor scores. Zaghni et al. [61] and Yilmaz et al. [59] stated that nurses were at a moderate level of emotional labor, Wu et al. [55] and Kwon et al. [44] found a moderate to high level, and Delgado et al. [31] and Lee and Ji [46] reported that nurses' emotional labor was at a high level.

In addition, inconsistent results were found in the description of nurses' emotional labor strategies. Cheng et al.'s [28] study identified hiding as the most common emotional labor strategy used by nurses, and Yilmaz et al. [59] also showed that nurses had the highest scores on superficial behaviors. However, Pooja and Bhoomadevi [51] and Yoon and Kim [60] found that nurse participants used deep acting strategies more frequently.

### 4.5. Relationships Between Emotional Labor and Outcomes

Our review identified significant associations between nurses' emotional labor and 52 outcomes categorized into two main themes: nurses' personal well-being and organizational well-being.  Table 3 demonstrates the significant outcomes related to nurses' emotional labor.

Thirty three studies focused on personal well-being, which was divided into burnout, satisfaction, and personal psychological states. Most studies have shown that emotional labor was negatively correlated with burnout and psychological states, and that high levels of emotional labor meant high burnout, high stress, high depression, and low resilience. However, some dimensions such as emotion regulation efforts [46], deep acting [32], and expression of natural emotions [32, 38] showed opposite results, as they were significantly negatively associated with burnout. Yilmaz et al.'s [59] study also showed a decrease in depression level among nurses as their emotional labor increased. In addition, an inconsistent relationship was found between emotional labor and job satisfaction. Almost all the studies have demonstrated a negative correlation between surface acting and job satisfaction and a positive relationship between deep acting and job satisfaction. However, Pooja and Bhoomadevi [51] suggested that surface acting had a significant positive effect on job satisfaction.

Seventeen outcomes were related to organizational well-being, including job performance and withdrawal behaviors. Firstly, eight studies showed a mixed relationship between emotional labor and nurses' job performance. Mauno et al. [11] suggested that higher emotional labor was associated with lower work engagement. However, the findings of Wang and Chang [54] and Kim and Jang [41] showed that higher emotional labor was related to higher customer-oriented behaviors and prosocial behaviors. A study showed that surface acting was negatively associated with organizational contribution [57], while on the contrary, Pooja and Bhoomadevi [51] demonstrated a positive effect of surface acting, with higher surface acting related to higher emotional involvement. Nine studies analyzed the relationship between emotional labor and nurses' withdrawal behaviors. Majority of studies have shown that higher emotional labor is associated with higher turnover intention [18, 20, 44, 73]. In addition, higher surface acting was associated with higher turnover intention [30], higher absenteeism [49], and higher presenteeism [53]. Higher deep acting was associated with lower turnover intention [30] and reduced presenteeism [53].

Finally, it is important to note that a study [33] examined the relationship between emotional labor and workplace violence among nurses, revealing that emotional labor could reduce workplace violence.

Among the included studies, we identified 19 mediators and 12 moderators between nurses' emotional labor and 26 outcomes. Stress (*n* = 4) was the most frequently tested mediator linking emotional labor to three different outcomes, including well-being, burnout, and emotional exhaustion. Three studies explored the mediating role of burnout between emotional labor and turnover intention and presenteeism. Two studies showed that the nurse-patient relationship mediated the relationship between emotional labor and nurses' burnout and job satisfaction. Several other significant mediators and moderators were only examined in individual studies, such as core self-evaluation, surface acting self-efficacy, emotional intelligence, resilience, affective commitment, leader-member exchange, perceived organizational commitment, and organizational support.

## 5. Discussion

### 5.1. The Level of Emotional Labor

When analyzing the level of the emotional labor of nurses, it is important to consider the differences between these measurement instruments. First, there were more than 10 different scales used for measurement in all of the included studies. Various instruments measure different dimensions. For example, the scale developed by Diefendorff [2] included surface acting, deep acting, and expression of naturally felt emotions. Morris and Feldman [63] focused on the need for emotional labor, which included frequency of emotional labor, attention level of emotional expression, and emotional dissonance. In Wang and Chang's study [54], the expression of positive emotions and the suppression of negative emotions were measured. Second, even though different studies used the same scale, the dimensions measured by the researchers also varied. For instance, the emotional labor scale developed by Brotheridge and Lee appeared to be described and used differently in five studies [28, 30, 31, 45, 50]. Pisaniello, Winefield, and Delfabbro [50] even used two scales simultaneously to measure nurses' emotional labor. In addition, many studies failed to provide adequate psychometric data about their instruments. Most studies provided Cronbach's alpha for the scales but did not report data related to validity. Four studies did not even report the score ranges or related psychometric data. This inconsistency made it difficult to compare the reported scores. It illustrates the insufficient integration of emotional labor theories in the field of nursing, particularly the absence of consistent operational concepts when measuring emotional labor.

### 5.2. Emotional Labor Outcomes

Our review integrated consequences associated with nurses' emotional labor, which not only confirmed Grandey's emotional labor model but also supplemented this theoretical framework. For example, we also identified individual mental health outcomes associated with emotional labor for nurses, including depression, anxiety, stress, and resilience. It was notable that we did not identify patient outcomes associated with nurses' emotional labor. Drach-Zahavy, Buchnic, and Granot [85] investigated the relationship between midwives' emotional labor strategies and the birth experience. The findings revealed that deep acting enhanced the maternal birth experience. This suggests that we could analyze the relationship between patient experience and nurses' emotional labor strategies, which could offer valuable information and guidelines for nurse managers and nursing practitioners to improve patient satisfaction.

The findings confirmed the complex outcomes of nurses' emotional labor. On the one hand, the emotional labor of nurses was found to increase burnout, decrease job satisfaction, impair mental health, and increase turnover intention. On the other hand, nurses' emotional labor yielded positive outcomes. It was able to decrease depression, increase job satisfaction, and even reduce workplace violence. This finding is consistent with the results of Yao et al. [58], a study showing the curvilinear effects of emotional labor. This phenomenon could be explained in the following three ways. First, emotional labor represents a nursing competence. Emotional role is a crucial aspect of the professional responsibilities of nurses. Emotional labor in healthcare settings can be defined as behaviors or skills required to engage in caring roles, identify others' emotions, and manage their own emotions [24]. Nurses not only experience strong emotions in their work but also consciously utilize these emotions to improve their practice [7]. The outcomes of emotional labor may depend on individuals' management skills in different situations. For example, many studies have shown that surface acting is associated with depression, low resilience, low work engagement, low job satisfaction, high burnout, and high turnover intention in nurses, while deep acting yields more positive results. Contrary to the hiding and suppression of surface acting, nurses who use deep acting consciously engage in emotional self-regulation and promote cognitive shifts to generate a genuinely empathic response. Therefore, nursing managers should pay attention to nurses' emotional demands and emotional regulation skills by providing emotional management training to encourage the use of deep acting strategies. Second, the Job Demands-Resources Model [86] also contributed to our understanding of the phenomenon, focusing on the interactions between work demands and work resources. Aspects of nursing interactions are part of emotional labor [87], and emotional labor in nursing contexts encompasses three types: therapeutic, instrumental, and collegial. Delgado et al. [23] reviewed emotional labor in nursing, which includes diversity in caring, dealing with negative emotions, caring for the terminally ill, and procedural nursing. It reflected the high emotional work demands of nursing. Surface acting and deep acting are the two most commonly used emotional labor strategies, which can deplete individuals' emotional resources. Comparatively, engaging in deep acting with emotion regulation can help nurses change their inner feelings, consume fewer emotional resources, and produce positive personal and organizational well-being. However, chronic emotional effort can deplete nurses' emotional resources and lead to negative outcomes. Finally, the complex outcomes may suggest the need for a deeper understanding of the process and contents of nurses' emotional labor. Unlike traditional theories, emotional labor in the context of healthcare settings has reciprocal value for both staff and patients [24]. Nurses' emotional labor is a genuine expression of compassion or a “gift” to the patient [5], which can provide excellent patient care and can be used to facilitate nurse-patient reciprocity. Smith and Gray also advocated that current models of emotional labor are limited in addressing the complexities associated with practice and management in the healthcare service [88]. This requires a new framework to provide a flexible theoretical basis.

Our findings also demonstrated mediators and moderators associated with nurses' emotional labor, providing insight into the mechanisms of emotional labor. This is a basis for nurse managers to improve nurses' satisfaction and decrease nursing turnover. First, at the individual level, emotional intelligence [17, 73], self-efficacy [49], stress [61], and core self-appraisal [8] played a key role. It is implied that nurse managers should pay attention to nurses' emotional experiences in order to reduce the negative effects of emotional labor on job satisfaction. At the same time, they should focus on enhancing the emotional intelligence of nurses to develop their emotional management skills and confidence. Nurse managers can provide emotional management training, such as emotional therapy and mindfulness, to help nurses acquire appropriate emotional regulation strategies, reduce stress level, improve the quality of care, and decrease burnout and turnover intentions. Second, from an organizational perspective, perceived high-performance work system [18], team climate [28], perceived organizational support [33, 40, 45], leadership-member exchange [46], and work ethic [11] were able to mitigate the negative effects of emotional labor. This also implies strategies for providing emotional resources and support to clinical nurses. Nurse managers can reduce nursing turnover by implementing high-performance work systems, creating a positive team climate and a healthy work environment, and providing positive organizational support. In addition, focusing on nurse managers' leadership skills and communication behaviors with team members can be beneficial in improving nurses' sense of organizational support in the workplace. Support groups could be established to facilitate the sharing of dilemmas and emotional experiences among nurses to improve their work ethic and shield them from the adverse effects of emotional labor.

## 6. Limitations

We must acknowledge some limitations of the scoping review. First, qualitative studies and gray literature were not included, and only studies in English were retrieved, which might lead to reporting bias. Second, except for two longitudinal studies, most studies were cross-sectional, which did not allow for causal inferences. Finally, the rules of emotional expression may differ across cultures. We should note that more than half of the original studies were from East Asia, which could be related to their collectivist culture. Eastern collectivistic cultures prioritize concern for others, and individuals are expected to manage their emotions and be sensitive to the emotions of others to promote harmony [89]. As a result, emotional expression tends to be more controlled in these cultures, with a strong emphasis on emotional regulation. This implies that future research may need to focus on the variations in emotional labor among nurses in different cultural contexts.

## 7. Conclusions

The findings of this review suggest that the emotional labor of nurses has received increasing attention, along with a growing body of related studies. The summary and analysis of the levels and outcomes of nurses' emotional labor advance this body of knowledge. Nurse managers can use the results of this review to recognize and value nurses' emotional labor and comprehend the individual and organizational outcomes. At the individual level, nurse managers can focus on nurses' emotional experiences and emotional intelligence. They can also provide training in emotional management to help nurses to deal with the diverse emotional demands of the workplace. At the organizational level, healthcare institutions and nurse managers need to provide positive organizational support and emotional resources for clinical nurses to reduce nursing turnover intentions.

## 8. Implications for Nursing Management

This review has important implications for nursing management. The findings can help nursing managers to have a better understanding of the level of nurses' emotional labor and its impact on both individuals and organizations. Nursing managers are advised to provide appropriate training and courses regularly to improve nurses' ability to manage emotions in patient interactions. In addition, it is an important step to explore different interventions to mitigate the negative effects of nurses' emotional labor.

Nurse managers and researchers need to pay attention to the following issues when researching emotional labor among nurses. First, it is important to select an emotional labor measurement instrument with good reliability and validity that is appropriate for healthcare workers. Second, it is necessary to explore the relationship between nurses' emotional labor and patient-related outcomes, for example, to assess whether different emotional labor strategies can be perceived by patients and how they may contribute to the experience of patients. Finally, focus on the positive effects of emotional labor. Exploring appropriate emotional labor from a positive perspective can help nurses to be more motivated and build trusting relationships with patients.

## Figures and Tables

**Table 1:** Summary characteristics of included studies.

**Author/country**	**Aim**	**Theoretical basis**	**Study design**	**Sample**	**Measurement outcomes**	**Data analysis**	**Recommendations**
Akkoç et al. [26]/Turkey	To investigate relationships between hospice nurses' emotional labor, life satisfaction, and affective commitment	Literature review	Cross-sectional study	322 nurses	Life satisfaction	Hayes' process	Nurse leaders should understand and transform the surface emotional setting of nurses to more profound acting emotions and then to natural emotional responses

Bartram et al. [18]/Australia	To explore the relationships between perceived high-performance work systems, emotional labor, burnout, and intention to leave among nurses	JD-R model	Cross-sectional study	183 nurses	Intention to leave	Baron and Kenny' s procedure and Saunders procedure	Ensuring effective human resource management practice through the implementation of high-performance work systems may reduce the burnout associated with emotional labor

Cha and Baek [27]/Korea	To investigate the factors influencing three dimensions of burnout among clinical nurses	Literature review	Cross-sectional study	300 nurses	Burnout	Multiple linear regression	Nurse managers should work with nursing organizations and government bodies to develop policies for a safe and healthy working environment to alleviate emotional exhaustion among nurses

Cheng et al. [28] /Australia	To examine the relationships among emotional labor, team climate, burnout, perceived quality of care, and turnover intention among nurses	The conservation of resources theory	Cross-sectional study	201 nurses	Burnout Quality of care Turnover intention	Structural equation model	Hospital managers need to devote more attention toward building a team climate among nurses to manage the emotional demands of their role

Chou, Hecker, and Martin [29]/(Taiwan, China)	To investigate the effects of job demands and resources as well as emotional labor on job satisfaction and emotional exhaustion among nurses	Literature review	Cross-sectional study	450 nurses	Job satisfaction Emotional exhaustion	Structural equation model	The outcomes of emotional labor can either be positive or negative. Organizations aiming to improve nurses' job satisfaction and diminish their emotional exhaustion should focus on training them in effective emotional regulation techniques and creating a climate in which nurses feel supported by their organization.

Cottinghama, Erickson, and Diefendorff [30]/USA	To shape the relationship between emotional labor and job satisfaction	Gender Frame Theory	Cross-sectional study	730 registered nurses A large Midwestern hospital	Job satisfaction Turnover intention	Multiple regression analyses	It is needed to assess the role of the nursing profession and workplace context in shaping male nurses' internalization of fewer emotional norms than their female colleagues to address gender inequality

Delgado et al. [31]/Australia	To explore the relationship between workplace resilience and emotional labor	Literature review	Cross-sectional study	482 mental health nurses	Resilience	General linear models	Clinical supervision may be a key strategy in mental health that could help manage the negative impacts of emotional labor and develop resilience at work

Deng et al. [32]/China	To examine the interactive effects of three emotional labor strategies and stress reactivity on job burnout	JD-R model	Cross-sectional study	229 nurses	Job burnout	Multiple linear regression analyses	High stress reactivity might be the plasticity factor for Chinese nurses' emotional exhaustion and depersonalization in the context of surface acting and deep acting, and low stress reactivity might be the plasticity factor for Chinese nurses' professional inefficacy in the context of the expression of naturally felt emotions

Erkutlu et al. [33]/Turkey	To investigate the relationship between emotional labor and workplace violence	The social exchange theory	Cross-sectional study	536 nurses	Workplace violence	Hierarchical multiple regression	Healthcare administrators should offer more training to nurses to help them manage their emotions while interacting with their patients. This leads to positive interpersonal relationships, which, in turn, lowers workplace violence.

Hong [34]/Korea	To identify emotional labor, job stress, emotional intelligence, and burnout as variables affecting nurses' turnover intention	Literature review	Cross-sectional study	211 nurses	Turnover intention	Structural equation model	As emotional labor's effect on turnover intention was mediated by job stress and burnout, nurse support programs should aim to reduce nurses' emotional labor in order to effectively reduce turnover intention

Hwang and Park [35]/Korea	To construct a structural equation model on clinical nurses' emotional labor, job satisfaction, and job performance	Grandey's emotional regulation model	Cross-sectional study	424 nurses	Job satisfaction Job performance	Structural equation model	The value of emotional labor is not properly evaluated and we must assign a value for emotional labor to the international scientific nursing to bolster nurse's self-esteem

Jung and Baek [36]/Korea	To investigate the effect of emotional labor and job stress on depression in nurses with long working hours	Literature review	Cross-sectional study	291 nurses Three general hospitals	Depression	Structural equation model	It is necessary to develop and apply programs that can promote resilience, as this could help reduce depression caused by emotional labor among nurses with long working hours

Karimi et al. [17]/Australia	To identify the relationships among emotional intelligence, emotional labor, well-being, and job stress in nurses	Emotion regulation theory	Cross-sectional study	312 community nurses	Well-being	Structural equation model	Findings suggest that on-the-job training and emotional intelligence training might be included in the structure of nursing courses and may assist in mitigating the serious effects ascribed to high levels of emotional labor

Kim et al. [37]/Korea	To confirm the relationships between clinical nurses' emotional labor, workplace violence, and burnout	Literature review	Cross-sectional study	356 nurses 4 university hospitals	Burnout	Structural equation model	Interventions aimed at reducing nurse burnout should include efforts to decrease emotional labor and workplace violence

Kim, Park, and Park [38]/Korea	To explore whether anger suppression and anger rumination mediate the relationship between emotional labor and depressive and anxiety symptoms of nurses	Literature review	Cross-sectional study	99 nurses A university hospital	Depression and anxiety	Sequential multiple mediation analysis	Constructing a specific intervention strategy to protect nurses' psychological adjustment is valuable, for example, training in the use of different kinds of adaptive emotional regulation strategies other than anger suppression and anger rumination

Kim [39]/Korea	To examine the associations among emotional labor strategies, stress from emotional labor, and burnout in nurses	Literature review	Cross-sectional study	303 nurses	Burnout	Structural equation model	Hospital organizations need to first identify how nurses perceive organizationally desired emotional labor as well as how nurses cope with the emotional labor. Providing interventions that improve nurses' ability to manage their emotions in interactions with patients might effectively reduce nurses' stress and burnout.

Kim et al. [40]/Korea	To identify the mediating effects of perceived health status and perceived organizational support in the association between emotional labor and burnout in public health nurses	Literature review	Cross-sectional study	207 nurses	Burnout	The parallel multiple mediator model	Interventions that provide effective emotional regulation management education, communication, and psychological counseling support to positively manage the negative effects of emotional labor

Kim and Jang [41]/Korea	To investigate the extent to which emotional labor influences Korean nurses' prosocial service behavior	Literature review	Cross-sectional study	145 nurses Korean tertiary hospitals	Prosocial service behavior	Multiple regression analysis	Nurses with an elevated level of emotional modulation seem to be better able to utilize their emotions during patient treatment. It is necessary to develop effective interventions to help them maintain this advantage.

Kumar and Jin [42]/China	To explore the relationship between job stress, emotional exhaustion, and surface acting	JD-R model Organizational support theory	Longitudinal study	319 frontline nurses 107 Pakistan government hospitals	Job stress and emotional exhaustion	Mediation analysis	Organizational support is essential as it may assist frontline nurses to deal with emotional exhaustion caused by emotional labor and stress

Kwak et al. [43]/Korea	To examine the impact of emotional labor and workplace violence on professional quality of life of clinical nurses	Literature review	Cross-sectional study	399 clinical nurses	Professional quality of life	Multiple regression	Organizational management systems that address and reduce emotional labor are critical in clinical settings

Kwon et al. [44]/Korea	To investigate the relationships between emotional labor, burnout, medical error, and turnover intention	Literature review	Cross-sectional study	117 nurses University-based general hospital	Medical error Turnover intention	Multiple regression analysis	The job positions of nursing personnel may be a major consideration in such a strategy, and job-focused emotional labor and employee-focused emotional labor may be promising targets in ameliorating turnover intention and client-related burnout, respectively

Kwon and Song [20]/Korea	To investigate the impact of emotional labor on the turnover intention of nurses	Literature review	Cross-sectional study	155 nurses	Turnover intention	Multivariable linear regression	To retain nurses, nurses and hospital managers should together implement programs aimed at reducing nurses' emotional labor and enhancing psychological capital program and increase the number of nurses

Lartey, Amponsah-Tawiah, and Osafo [45]/Ghana	To establish the link between emotional labor and job attitudes	JD-R model	Cross-sectional study	342 nurses and midwives Three general hospitals, two polyclinics, and one psychiatric hospital	Job satisfaction Organizational commitment	Hierarchical multiple regression	Nursing education and practice should pay attention to equipping students and professionals in terms of how best to handle the emotional demands of the profession as this has been found to have bearing on their job attitudes

Lee and Ji [46]/Korea	To identify the moderating effect of leader-member exchange in the relationship between emotional labor and burnout among clinical nurses	Literature review	Cross-sectional study	165 nurses	Burnout	Three-step hierarchical multiple regression	Strengthening emotional modulation efforts in the profession while reducing patient-focused emotional suppression can help reduce burnout among nurses

Lee and Jang [47]/Korea	To examine the relationships between emotional labor, emotions, and job satisfaction among nurses	Literature review	Cross-sectional study	168 nurses	Job satisfaction	Structural equation model	It needs practical interventions to inform nurses and managers as well as administrators of the beneficial impacts of deep acting and of the potentially detrimental influences of surface acting, and to promote the use of deep acting instead of surface acting among nurses

Liu et al. [48]/China	To test the mediating role of the nurse-patient relationship and self-rated health in the effect of emotional labor on turnover intention among nurses	Literature review	Cross-sectional study	527 nurses	Turnover intention	Baron and Kenny's causal steps and the Karlson/Holm/Breen method	While emotional labor is inherently intertwined with the nursing profession, it is imperative to recognize and address the potential individual, institutional, and societal repercussions linked to emotional labor

Mauno et al. [11]/Finland	To examine whether compassion, transformational leadership, and work ethic feasibility buffer against the negative effects of emotional labor on work engagement	JD-R model	Cross-sectional study	3466 nurses	Work engagement	Moderated hierarchical regression analysis	We recommend that when nursing organizations want to improve their staff's work engagement, they need to decrease emotional labor to help their employees to cope better with the demands of emotional labor

Nguyen, Groth, and Johnson [49]/Australia	To examine absenteeism as a key long-term consequence of emotional labor	Conservation of resources theory	Longitudinal study	121 nurses	Absenteeism	Hierarchical linear regression equations	Training employees to be confident with managing their emotional displays might have some beneficial effects, such as reducing absenteeism

Pisaniello, Winefield and Delfabbro [50]/Australia	To investigate the relationship between emotional labor and emotional work on psychological well-being and occupational stress	Conservation of resources theory	Cross-sectional study	239 nurses A large metropolitan public hospital	Stress Burnout Job satisfaction	Hierarchical multiple regression	Psychosocial factors such as the companionship, positive family-work, positive spillover, and supervisor social support may offset the effects of negative outcomes

Pooja and Bhoomadevi [51]/India	To assess emotional labor and its outcomes among nurses	Literature review	Cross-sectional study	270 nurses	Positive outcomes Negative outcomes	Multiple regression	It is important to prioritize nurses' emotional well-being, promote authentic emotional expression, and develop strategies to mitigate the potential negative outcomes associated with emotional labor

Saei, Sarshar, and Lee [8]/Iran	To investigate the mediating and moderating impact of core self-evaluations in the path from emotional labor to burnout	The conservation of resources theory	Cross-sectional study	255 nurses	Burnout	Structural equation model Hierarchical moderated regression	Capacity as mediator and moderator for nurses in the midst of the pandemic. Managing the demands of emotional labor through this trait will enhance clinical care and well-being for both patients and caregivers.

Schmidt and Diestel [52]/Germany	To investigate the relationship between emotional labor strategies and cognitive control deficits with job strain	The strength model of self-control Conservation of resources theory	Cross-sectional study	195 nurses A hospital and three nursing homes	Job strain	Hierarchical moderated regression analyses	Promote deep acting instead of surface acting and strengthen nurses' control capacity

Song et al. [53]/China	To examine the correlations between emotional labor, burnout, and presenteeism	JD-R model Emotional Labor theory	Cross-sectional study	1038 nurses 6 tertiary hospitals	Presenteeism	Structural equation model	Self-training mindfulness, emotion therapy, or other effective methods could improve the emotional management skills of the nurses and help them to alleviate burnout and reduce presenteeism

Wang and Chang [54]/(Taiwan, China)	To explore the relationships among emotional labor, job involvement, and customer-oriented behavior in the context of the nursing profession	Literature review	Cross-sectional study	472 nurses	Customer-oriented behavior	Hierarchical regression analysis	For the benefit of both employees and customers, employees should be able to successfully manage their emotions. They should not feel the need to inhibit their emotions nor express insincere emotions

Wu et al. [55]/China	To investigate the associations of emotional labor and core competency with job satisfaction	Literature review	Cross-sectional study	13448 nurses from 92 hospitals	Job satisfaction	Multiple linear regression	Nurses' deep acting of their emotions should be improved and surface acting should be managed appropriately Nursing courses and professional training programs should increase focus on the following to deal with the emotional labor of the nursing profession more efficiently

Xu and Fan [56]/China	To investigate the relationships among emotional labor, nurse-patient relationship, and job satisfaction	Healthcare model of emotional labor	Cross-sectional study	496 nurses	Job satisfaction	Structural equation model	Strategies can be employed to counteract the negative effects of emotional labor performance. In addition, more opportunities should be provided to discuss the use of different strategies for emotional labor and encourage appropriate responses based on the patient's situation and work context.

Yang and Chang [57]/(Taiwan, China)	To examine the relationship between emotional labor, job satisfaction, and organizational commitment	Literature review	Cross-sectional study	295 nurses	Job satisfaction Organizational Commitment	Structural equation model	When nurses perform emotional labor that differs from their inner feelings, it will not affect their degree of job satisfaction but depress their organizational commitment. When emotional labor coincides with inner feelings, job satisfaction is enhanced but the effect on organizational commitment is negligible.

Yao et al. [58]/China	To examine if there was a curvilinear relationship between emotional labor and work engagement in Chinese nurses	Motivation theory JD-R model	Cross-sectional study	528 nurses	Work engagement	Curvilinear regression model	Nurses should learn to regulate emotions and engage in emotion labor appropriately in clinical practice so as to exert the positive effects of emotional labor to a greater extent

Yilmaz et al. [59]/Turkey	To investigate the effects of emotional labor and secondary traumatic stress in midwives and nurses on their depression levels	Literature review	Cross-sectional study	313 participants (231 nurses 82 midwives)	Depression	Structural equation model	It is needed to improve working conditions, increase the level of social support, and mobilize strategies of coping with stress to prevent problems regarding emotional labor and desensitization

Yoon and Kim [60]/USA	To examine the relationship between job-related stress, emotional labor, and depressive symptoms	Literature review	Cross-sectional study	441 nurses Five general hospitals	Depressive symptoms	Multivariate logistic regression	Programs for nurses need to be created that will help reduce expectations for surface acting and control job-related stress, thus preventing the development of depressive symptoms

Zaghini et al. [61]/Italy	To evaluate the influence of emotional labor on burnout and the mediating role of work-related stress	Literature review	Cross-sectional study	207 nurses from three hospitals	Burnout	Structural equation model	The results of this study highlight the mediating role of work-related stress in the relationship between emotional labor and burnout, offering a new field for intervention to interrupt this process

**Table 2:** Instrument characteristics and results.

**Author/country**	**Scale to measure emotional labor**	**Response scale range**	**Reliability/validity**	**Dimension**	**EL scores (mean ± SD)/M (P25, P75)**
Akkoç et al. [26]/Turkey	The emotional labor scale [2]	1 (strongly disagree) to 5 (strongly agree)	Not mentioned	Surface acting Deep acting Expression of naturally felt emotions	2.28 ± 0.89 3.14 ± 1.03 3.26 ± 1.05

Bartram et al. [18]/Australia	Seven-item scale comprising negative and positive efference items [62]	Not mentioned	*α* = 0.77/not mentioned	Not mentioned	Total: 3.44 ± 0.64

Cha and Baek [27]/Korea	The emotional labor scale [63]	1 (strongly disagree) to 5 (strongly agree)	*α* = 0.86/not mentioned	Frequency of emotional labor Attention level of emotional expression Emotional dissonance	Total: 32.15 ± 6.21

Cheng et al. [28]/Australia	The modified version of the emotional labor scale [64]	1 (never) to 5 (always)	Not mentioned	Surface acting-faking Surface acting-hiding Deep acting	7.27 ± 2.46 9.51 ± 1.91 8.63 ± 2.34

Chou, Hecker, and Martin [29]/(Taiwan, China)	The emotional labor scale [2]	1 (never) to 5 (always)	*α* = 0.82 to 0.91/CFA	Surface acting Deep acting	2.33 ± 0.91 3.24 ± 0.87

Cottinghama, Erickson, and Diefendorff [30]/USA	The emotional labor scale [65]	1 (never) to 5 (almost always)	*α* = 0.84 to 0.93/not mentioned	Surface acting-cover Surface acting-pretend Deep acting	Woman man 2.96 ± 0.68 2.8 ± 0.67 2.52 ± 0.81 2.24 ± 0.73 2.77 ± 0.81 2.52 ± 0.82

Delgado et al. [31]/Australia	The emotional labor scale [65]	1 (never) to 5 (always)	*α* = 0.81/not mentioned	Duration Frequency Intensity Variety Surface acting Deep acting	32 ± 22.7 min 10.83 ± 1.81 5.29 ± 1.41 9.50 ± 2.02 8.87 ± 2.05 9.31 ± 2.41

Deng et al. [32]/China	The emotional labor strategy scale [66]	1 (strongly disagree) to 5 (strongly agree)	*α* = 0.76 to 0.86/not mentioned	Surface acting Deep acting Expression of naturally felt emotions	2.59 ± 0.65 3.13 ± 0.63 3.44 ± 0.74

Erkutlu et al. [33]/Turkey	Emotional labor scale for nurses [67]	1 (not at all) to 5 (very true)	*α* = 0.83/not mentioned	Not mentioned	Total: 3.34 ± 1.07

Hong [34]/Korea	The emotional labor scale [68]	1 (not mentioned) to 5 (not mentioned)	*α* = 0.84/not mentioned	Frequency of emotional display Attentiveness to required display rules Emotional dissonance	3.8 ± 0.57 3.6 ± 0.64 3.4 ± 0.65 Total: 3.6 ± 0.53

Hwang and Park [35]/Korea	The emotional labor scale [69]	1 (very high) to 5 (very low)	*α* = 0.89/not mentioned	Surface acting Deep acting	3.48 ± 0.594 3.29 ± 0.64

Jung and Baek [36]/Korea	The emotional labor tool [68]	1 (not mentioned)to 5 (not mentioned)	*α* = 0.625 to 0.772/not mentioned	Frequency of emotional display Attentiveness to required display rules Emotional dissonance	3.49 ± 0.77 3.39 ± 0.72 3.23 ± 0.75

Karimi et al. [17]/Australia	Scale developed by [70]	1 (very rarely/never) to 5 (very often)	*α* = 0.77/not mentioned	Emotional dissonance	2.888 ± 0.67

Kim et al. [37]/Korea	The Korean emotional labor scale [71]	1 (never) to 4 (often)	*α* = 0.792 to 0.815/not mentioned	Emotional demands and regulation Overload and conflict in customer service Emotional disharmony and harm	Total: 69.98 ± 14.75

Kim, Park, and Park [38]/Korea	The Korean emotional labor scale [71]	1 (strongly disagree) to 4 (strongly agree)	*α* = 0.87/not mentioned	Not mentioned	Total: 69.31 ± 9.07

Kim [39]/Korea	Korean version of the nurse emotional labor strategy scale [72]	1 (strongly disagree) to 5 (strongly agree)	*α* = 0.81 to 0.89/not mentioned	Surface acting Deep acting Naturally felt emotions	24.48 ± 4.49 21.92 ± 4.98 9.21 ± 1.89

Kim et al. [40]/Korea	The emotional labor scale [63]	1 (not at all) to 5 (very much so)	*α* = 0.88/not mentioned	Frequency of emotional labor Attention level of emotional expression Emotional dissonance	Total: 31.55 ± 5.68

Kim, Park, and Park [38]/Korea	The emotional labor scale for nurses [73]	1 (not at all) to 5 (very much)	*α* = 0.81/CFA	Emotional control efforts in profession Patient-focused emotional suppression Emotional pretense by norms	3.75 ± 0.45 3.24 ± 0.52 3.07 ± 0.70 Total: 3.41 ± 0.48

Kumar and Jin [42]/China	The emotional labor scale [65]	Not mentioned	*α* = 0.72/CFA	Surface acting (only measured)	5.12 ± 1.25

Kwak et al. [43]/Korea	The Korean emotional labor scale [71]	1 (never) to 4 (often)	*α* = 0.737 to 0.751/not mentioned	Magnitude of exposure to emotional labor Organizational management systems for emotional labor	212.2 ± 44.5 110.2 ± 29.1

Kwon et al. [44]/Korea	The emotional labor assessment tool of Lee [74]	1 (not at all) to 5 (very much)	*α* = 0.71/not mentioned	Employee-focused emotional labor Job-focused emotional labor	3.48 ± 0.54 3.50 ± 0.51

Kwon and Song [20]/Korea	Emotional labor scale for nurses [73]	1 (not at all) to 5 (very much)	*α* = 0.87/not mentioned	Emotional modulation efforts in the profession Patient-focused emotional suppression Emotional pretense by norms	Total: 54.52 ± 7.55

Lartey, Amponsah-Tawiah, and Osafo [45]/Ghana	The emotional labor scale [65]	Not mentioned	*α* = 0.71 to 0.90/not mentioned	Surface acting Deep acting	7.26 ± 2.78 9.51 ± 2.81

Lee and Ji [46]/Korea	The emotional labor scale for nurses [73]	1 (not at all) to 5 (very much)	*α* = 0.76 to 0.91/not mentioned	Emotional control efforts in profession Patient-focused emotional suppression Emotional pretense by norms	25.83 ± 3.32 17.30 ± 3.65 13.14 ± 2.47

Lee and Jang [47]/Korea	Korean version of emotion labor strategies scale [75]	1 (never) to 5 (always)	*α* = 0.71 to 0.78/not mentioned	Surface acting Deep acting	3.11 ± 0.48 3.21 ± 0.51

Liu et al. [48]/China	The Chinese version of the emotional labor scale [76]	1 (strongly disagree) to 5 (strongly agree)	*α* = 0.876/not mentioned	Variety Intensity Duration Frequency of emotional display Surface acting Deep acting	Total: 45.23 ± 9.12

Mauno et al. [11]/Finland	Two dimensions in a large validation study [77]	1 (rarely, never) to 5 (very often, always)	*α* = 0.61/not mentioned	Emotional dissonance Emotional sensitivity requirements at work	Total: 3.37 ± 0.5

Nguyen, Groth, and Johnson [49]/Australia	The emotional labor scale [78]	1 (not at all) to 5 (a great extent)	Not mentioned	Surface acting (only)	2.12 ± 0.089

Pisaniello, Winefield, and Delfabbro [50]/Australia	The emotional labor scale [65]	1 (never) to 5 (always)	*α* = 0.74 to 0.91/not mentioned	Duration Surface acting Deep acting (three subscales were used)	/ 8.22 ± 4.67 8.53 ± 3.77

Pisaniello, Winefield, and Delfabbro [50]/Australia	The emotion work requirements scale [79]	1 (not at all) to 5 (always required)	*α* = 0.76 to 0.77/not mentioned	Display positive emotions Hide negative emotions	14.42 ± 2.8 11.3 ± 3.24

Pooja and Bhoomadevi [51]/India	Emotional labor scale for nurses [67]	not mentioned	*α* = 0.843/not mentioned	Surface acting Deep acting	(2.44 to 4.20) ± (0.97 to 1.27) (2.59 to 4.09) ± (1.01 to 1.42)

Saei, Sarshar, and Lee [8]/Iran	The emotional labor scale [65]	1 (rarely or never) to 5 (always or most often)	not mentioned	Surface acting Deep acting	11.84 ± 4.01 11.94 ± 5.18

Schmidt and Diestel [52]/Germany	The emotional labor scale [65]	1 (never) to 5 (always)	*α* = 0.68 to 0.86/not mentioned	Surface acting Deep acting	2.61 ± 0.71 2.94 ± 0.81

Song et al. [53]/China	The Chinese version of the nurse emotional labor strategy scale [80]	1 to 6	*α* = 0.70 to 0.85/not mentioned	Surface acting Deep acting Emotionally expressed demands	Total: 51.38 ± 9.72

Wang and Chang [54]/(Taiwan, China)	The improved version of the emotional labor scale [81]	1 (strongly disagree) to 6 (strongly agree)	*α* = 0.91 to 0.92/EFA	The expression of positive emotion The suppression of negative emotion	4.56 ± 0.99 4.52 ± 0.98

Wu et al. [55]/China	The Chinese version of nurse emotional labor questionnaire [80]	1 (extremely disagree) to 6 (extremely agree)	*α* = 0.711 to 0.872/not mentioned	Surface acting Deep acting Requirement to express emotions	3.52 ± 0.92 4.95 ± 0.81 3.91 ± 1.02 Total: 55.08 ± 9.63

Xu and Fan [56]/China	The Chinese version of the emotional labor scale [66]	1 (not mentioned) to 5 (not mentioned)	*α* = 0.71 to 0.75/not mentioned	Surface acting Deep acting Naturally felt emotions	3.13 ± 0.96 3.80 ± 0.73 3.52 ± 0.68

Yang and Chang [57]/(Taiwan, China)	Modified emotional labor loading scale [82]	1 (strongly disagree) to 7 (strongly agree)	*α* = 0.75 to 0.89/not mentioned	Emotional display rule Surface acting Deep acting Variety of emotions required Frequency and duration of interactions	5.46 ± 1.38 5.60 ± 1.05 6.41 ± 0.57 3.91 ± 1.42 5.56 ± 1.03

Yao et al. [58]/China	The emotional labor scale for nurses [83]	1 (not at all) to 5 (very much)	*α* = 0.764 to 0.881/EFA	Emotional control efforts in profession Patient-focused emotional suppression Emotional pretense by norms	4.29 (3.86, 4.71) 3.60 (3.00, 4.20) 3.50 (3.00, 4.00) Total: 3.94 (3.63, 4.19)

Yilmaz et al. [59]/Turkey	The emotional labor scale [64]	1 (absolutely disagree) to 5 (absolutely agree)	*α* = 0.82/not mentioned	Surface behavior Deep behavior Natural behavior	13.42 ± 5.3 12.33 ± 4.66 10.51 ± 3.79 Total: 36.26 ± 9.84

Yoon and Kim [60]/USA	The emotional labor scale [74]	1 (never) to 5 (always)	*α* = 0.64 to 0.791/not mentioned	Surface acting Deep acting	3.0 ± 0.6 3.5 ± 0.6

Zaghini et al. [61]/Italy	The emotional labor scale [84]	1 (never) to 5 (always)	*α* = 0.81/not mentioned	Surface acting Restraint Compliance	Total: 2.64 ± 0.62

**Table 3:** Outcomes associated with emotional labor according to Grandey's framework.

**Outcomes from Grandey's framework**	**Outcomes**	**Findings**	**Mediating factor(s)**	**Moderating factor(s)**	**Supporting studies**
Individual well-being	Well-being	Emotional labor had a negative direct and indirect effect on nurses' well-being Nurses with high emotional intelligence are less affected and experience less job stress and in turn experience better well-being	Job stress	Emotional intelligence	Karimi et al. [17]/Australia
	Well-being	Surface acting and deep acting had a significant effect on emotional exhaustion, negative emotions, and stress and job exhaustion	/	/	Pooja and Bhoomadevi [51]/India
	Burnout	Employee-focused emotional labor significantly positively impacted total burnout	/	/	Kwon et al. [44]/Korea
	Burnout	Higher EL associated with higher burnout directly and indirectly	Workplace violence	/	Kim et al. [37]/Korea
	Burnout	Emotional modulation efforts in the profession were negatively correlated with burnout and the effect was strengthened by leader-member exchange Patient-focused emotional suppression was positively correlated with burnout and the effect was weakened by leader-member exchange	/	Leader-member exchange	Lee and Ji [46]/Korea
	Burnout	Surface acting contributed to work-related burnout and patient-related burnout Deep acting contributed to poor patient-related burnout Suppressing negative emotion is a risk factor for personal burnout	/	/	Pisaniello, Winefield, and Delfabbro [50]/Australia
	Burnout	Hiding was positively related to burnout	/	/	Cheng et al. [28]/Australia
	Burnout	Emotional labor raised burnout directly and indirectly	Perceived health status Perceived organizational support	/	Kim et al. [40]/Korea
	Job burnout	Surface acting could positively predict emotional exhaustion, depersonalization, personal burnout, work-related burnout, and client-related burnout Deep acting could negatively predict all six aspects of job burnouts Expression of naturally felt emotion could also negatively predict all six aspects of job burnouts	/	Hair cortisol	Deng et al. [32]/China
	Burnout	Surface acting had a positive indirect effect on burnout Expression of naturally felt emotions had a negative direct effect on burnout	Stress from emotional labor	/	Kim [39]/Korea
	Burnout	Emotional labor had a positive direct and indirect effect on burnout	Stress	/	Zaghini et al. [61]/Italy
	Burnout	Surface acting has direct and positive effects on burnout Deep acting has indirect effects on burnout through core self-evaluations Core self-evaluations moderate the impact of surface acting on burnout	Core self-evaluations	Core self-evaluations	Saei, Sarshar, and Lee [8]/Iran
	Burnout	Emotional labor positively correlated with burnout	/	/	Cha and Baek [27]/Korea
	Emotional exhaustion	Surface acting affected emotional exhaustion positively	/	/	Chou, Hecker, and Martin [29]/(Taiwan, China)
	Emotional exhaustion	Surface acting had a positive indirect relationship with emotional exhaustion	Job stress in emergency	/	Kumar and Jin [42]/China
	Job satisfaction	Surface acting affected job satisfaction negatively Deep acting affected job satisfaction positively	/	/	Chou, Hecker, and Martin [29]/(Taiwan, China)
	Job satisfaction	Surface acting was negatively related to job satisfaction Deep acting was positively related to job satisfaction	/	/	Wu et al. [55]/China
	Job satisfaction	Surface acting negatively predicted job satisfaction directly and indirectly Deep acting positively predicted job satisfaction directly and indirectly Naturally felt emotions positively predicted job satisfaction indirectly	Nurse-patient relationship	/	Xu and Fan [56]/China
	Job satisfaction	More surface acting correlated with lower job satisfaction More deep acting correlated with better job satisfaction	/	/	Hwang and Park [35]/Korea
	Job satisfaction	Suppressing negative emotion is a risk factor for job dissatisfaction	/	/	Pisaniello, Winefield, and Delfabbro [50]/Australia
	Job satisfaction	Deep acting was positively related to job satisfaction for men Covering up felt emotions was associated with lower levels of job satisfaction among female nurses	/	/	Cottinghama, Erickson, and Diefendorff [30]/USA
	Job satisfaction	Surface acting related negatively with job satisfaction and the effect was weakened by perceived organizational commitment	/	Perceived organizational commitment	Lartey, Amponsah-Tawiah, and Osafo [45]/Ghana
	Job satisfaction	Deep acting correlated positively with job satisfaction Enjoyment, pride, and anger mediated the relationship between deep acting and job satisfaction	Emotions	/	Lee and Jang [47]/Korea
	Job satisfaction	Deep acting significantly and positively correlated with job satisfaction Frequency and duration interaction was significantly and negatively correlated with job satisfaction	/	/	Yang and Chang [57]/(Taiwan, China)
	Job satisfaction	Surface acting and deep acting had a significant positive effect on job satisfaction	/	/	Pooja and Bhoomadevi [51]/India
	Life satisfaction	Deep acting had a positive direct and indirect effect on life satisfaction Expression of naturally felt emotions had a positive direct and indirect effect on life satisfaction	Affective commitment	Affective commitment	Akkoç et al. [26]/Turkey
	Professional quality of life	Compassion satisfaction was likely to increase when organizational management systems for emotional labor were higher Burnout was associated with exposure to emotional labor	/	/	Kwak et al. [43]/Korea
	Depression and anxiety	Emotional labor had an indirect sequential effect on depressive and anxiety symptom	Anger suppression Anger rumination	/	Kim, Park, and Park [38]/Korea
	Depression	As the emotional labor scores increased, their depression scores decreased	/	/	Yilmaz et al. [59]/Turkey
	Depression	Emotional labor had a positive direct effect on depression	Resilience	/	Jung and Baek [36]/Korea
	Depressive symptoms	Surface acting was the second strongest contributor to an increased risk for depressive symptoms	/	/	Yoon and Kim [60]/USA
	Stress	Surface acting and suppressing negative emotion contributed to stress	/	/	Pisaniello, Winefield, and Delfabbro, [50]/Australia
	Job strain	Surface acting related positively with exhaustion, depressive symptoms, and sum of days absent. And the effect was strengthened by control deficits.	/	Control deficits	Schmidt and Diestel [52]/Germany
	Resilience	Higher surface acting and intensity associated with lower resilience Higher frequency associated with higher resilience	/	/	Delgado et al. [31]/Australia

Organizational well-being	Job performance	More deep acting correlated with better job performance	/	/	Hwang and Park [35]/Korea
	Work behaviors	Surface acting and deep acting had a significant effect on emotional involvement in job, kindness in treating patients, fulfilling patient expectations, gaining patient's interest, and relationship building with patient	/	/	Pooja and Bhoomadevi [51]/India
	Organizational commitment	Surface acting had a significantly negative relationship with organizational commitment Deep acting raised organizational commitment through job satisfaction	Job satisfaction	/	Yang and Chang [57]/(Taiwan, China)
	Work engagement	Higher emotional labor was directly associated with lower work engagement	/	Compassion Work ethic feasibility	Mauno et al. [11]/Finland
	Work engagement	The higher the degree of professional emotional control, the higher the level of nurse's work engagement An inverted U-shaped curve relationship was found between patient-focused emotional suppression and work engagement An inverted U-shaped curve relationship was found between emotional pretense by norms and work engagement	/	/	Yao et al. [58]/China
	Customer-oriented behavior	The expression of positive emotion has a positive effect on patient-oriented customer-oriented behavior and task-oriented customer-oriented behavior The suppression of negative emotion has a positive effect on patient-oriented customer-oriented behavior	/	Job involvement	Wang and Chang [54]/(Taiwan, China)
	Prosocial service behavior	As emotional labor increases, prosocial service behavior increases accordingly	/	/	Kim, Park, and Park [38]/Korea
	Quality of care	Faking was negatively related to perceived quality of care	/	/	Cheng et al. [28]/Australia
	Turnover intention	Deep acting was negatively related to turnover intentions for men Covering up felt emotions was associated with a higher turnover intention among female nurses	/	/	Cottinghama, Erickson, and Diefendorff [30]/USA
	Turnover intention	Hiding had positive effects on turnover intention directly and indirectly	Burnout	/	Cheng et al. [28]/Australia
	Turnover intention	Emotional labor significantly indirectly affected turnover intention through job stress and burnout	Emotional intelligence Burnout	/	Hong [34]/Korea
	Turnover intention	Emotional labor was positively associated with turnover intention Self-rated poor health and a disharmonious nurse-patient relationship partially mediated the positive effect of emotional labor on turnover intention	nurse-patient relationship	/	Liu et al. [48]/China
	Turnover intention	Job-focused emotional labor had a significant positive impact on turnover intention	/	/	Kwon et al. [44]/Korea
	Turnover intention	Emotional labor had a positive and significant effect on turnover intention	/	/	Kwon and Song [20]/Korea
	Intention to leave	Emotional labor has an indirect effect on intention to leave via burnout High-performance work systems reduce the adverse effects of emotional labor on burnout	Burnout	High-performance work systems	Bartram et al. [18]/Australia
	Presenteeism	Deep acting alleviated reduced presenteeism directly and indirectly Emotionally expressed demands raised presenteeism directly and indirectly Surface acting raised presenteeism indirectly	Burnout	/	Song et al. [53]/China
	Absenteeism	Nurses who reported engaging in more surface acting had higher absenteeism	/	Surface acting self-efficacy	Nguyen, Groth, and Johnson [49]/Australia

Others	Workplace violence	Emotional labor has negative effect on workplace violence directly and indirectly Nurses with weaker organizational support had a higher relationship between emotional labor and exposure to workplace violence	Patient-positive treatment	Organizational support	Erkutlu et al. [33]/Turkey

## Data Availability

Data sharing is not applicable to this article as no new data were created or analyzed in this study.

## Supporting Information

Search strategies used for PubMed associated with this article can be found in supporting data.
