# Supplementary material for: The Level and Outcomes of Emotional Labor in Nurses: A Scoping Review
Source: J Nurs Manag. 2024 Oct 15;2024:5317359. doi: 10.1155/2024/5317359 (PMC11919068; doi:10.1155/2024/5317359)
Supplement: Supporting Information — Additional supporting information can be found online in the Supporting Information section. [file 5317359.f1.docx]

**Appendix A. Supplementary Data**

Search strategies used for Pubmed

1. emotional labor
2. emotional labour
3. emotional work
4. 1 or 2 or 3
5. nurs*
6. Nurses[Mesh]
7. 5 or 6
8. 4 and 7
9. Filters: Publication date to current(20240312); Language: English

Result：333
